# Effects of hydrogen peroxide, temperature and treatment time on degradation properties of polyethersulfone ultrafiltration membrane

**DOI:** 10.3906/kim-2109-10

**Published:** 2021-10-05

**Authors:** Yasin ÖZAY, Erdal YABALAK, Nadir DİZGE

**Affiliations:** 1 Tarsus University, Department of Environmental Protection Technologies, Mersin, Turkey; 2 Department of Chemistry, Mersin University, Mersin, Turkey; 3 Department of Environmental Engineering, Mersin University, Mersin, Turkey

**Keywords:** Chemical cleaning, membrane degradation, polyethersulfone ultrafiltration membrane, hydrogen peroxide, H_2_O_2_, temperature

## Abstract

Oxidative cleaning agents such as hydrogen peroxide (H_2_O_2_) and sodium hypochlorite (NaClO) used in water and wastewater treatment play an important role in the degradation and rapid aging of the polymeric membranes. In addition, when the temperature is above the maximum operating range of the membrane, it negatively affects the membrane performance. H_2_O_2_, which is also known as a green and environmentally friendly strong oxidant because of releasing only water as a by-product, can provide good cleaning efficiency under temperature, but its influence on membrane aging is not fully understood. In this study, the aging of polyethersulfone (PES) ultrafiltration (UF) membrane using H_2_O_2_ under high-temperature conditions and degradation of the polymeric membrane were systematically investigated using response surface methodology (RSM). The effects of H_2_O_2_ concentration, temperature, and treatment time were tested on membrane flux, contact angle, pore size, and porosity for decomposed membrane. The results showed that normalized permeability was significantly changed approximately 2.34-folds by H_2_O_2_ concentration at an exposure dose of 5 mM and 373 K temperature. Moreover, the largest pore sizes as 161.23 nm and 160.73 nm were obtained at the conditions of 2.5 mM H_2_O_2_ concentration and 373 K temperature. The lowest contact angle (54.76°) and porosity (61.88%) were obtained at the same conditions. The results depicted that H_2_O_2_ can be used for membrane cleaning with minimum membrane degradation at moderate conditions.

## 1. Introduction

Ultrafiltration (UF) membranes are a widely used accepted technology for water and wastewater treatment due to their excellent rejection capabilities against particles and pathogens as well as acceptable capital and operating costs [1,2]. However, the biggest obstacle to the wide application of these membranes is membrane contamination, which is an inherent disadvantage of the membrane [3]. Physically irreversible fouling is still unavoidable during long-term operation although membrane fouling can be mitigated by various strategies such as pretreatment of feed water optimization of operating parameters, and the development of antifouling membranes [4]. Therefore, chemical cleaning is vital for the sustainable operation of the UF system [5]. Many chemicals are used as membrane cleaning agents. For example acids, bases, oxidants, surfactants, and complexing agents can be used for membrane cleaning [6]. Among these cleaning agents, oxidative substances such as hydrogen peroxide (H_2_O_2_) are widely used in membrane cleaning due to their high cleaning efficiency for organic and biological contamination, which are the main types of fouling in UF membranes used in water and wastewater treatment [5,7].

Hydrogen peroxide (H_2_O_2_) is a strong oxidant used in the chemical cleaning of membranes with a standard reduction potential of 1.78 V. However, its reactivity is limited by a relatively high activation energy barrier [8,9]. The effectiveness of H_2_O_2_ has been demonstrated in several membrane studies for permeability recovery [10,11]. It was reported that H_2_O_2_ cleaning under strong alkaline conditions can supply higher cleaning efficacy compared to NaClO for chemical cleaning of UF membrane fouled by humic substances [8]. It was also reported that H_2_O_2_ cleaning prevented the formation of toxic halogenated by-products compared to cleaning with NaClO. Hence, H_2_O_2_ can be considered a potential alternative cleaning agent to commonly used NaClO [6]. However, although numerous articles have been published using H_2_O_2_ as a cleaning chemical, only a few papers are investigating the aging of the polymeric membrane by H_2_O_2_ [7]. Ling et al. [12]. investigated the tolerance of a thin-film composite polyamide reverse osmosis membrane to H_2_O_2_ exposure. Yu et al. [13]. focused on the iron-catalyzed degradation of a polyamide nanofiltration membrane by H_2_O_2_. The effects of H_2_O_2_ enhanced backwashing on the mechanical properties and surface functional groups of PVDF membrane were examined for prevention of membrane fouling in drinking water treatment [14]. In general, the aging of the UF PES membrane due to H_2_O_2_ cleaning is poorly understood.

The main purpose of this study was to comprehensively investigate the degradation of the PES membrane caused by H_2_O_2_, temperature, and treatment time. Firstly, the PES membrane was fouled by methylene blue (MB) dye (100 mg/L) at 1 bar. Secondly, fouled PES membrane was cleaned by H_2_O_2_ under temperature conditions. Response surface methodology (RSM) was used to systematically investigate the effect of H_2_O_2,_ temperature, and treatment time on membrane cleaning and degradation of PES membrane. Apart from being a statistical design method that is frequently preferred by researchers, RSM enables evaluation of the interaction of variables affecting the system in many experimental procedures and revealing the effects of these parameters on the response, thus providing reactive, labor, and time savings by making effective optimization [15,16]. RSM designs are superior to single factor analysis methods (one-factor-at-a-time) in many aspects such as the ability to effectively evaluate the relationship between variables and the optimization of the system, requiring relatively few experiments, and deriving hypothetical mathematical equations of the response [17,18]. The Box-Behnken Design (BBD) is one of the widely used RSM designs and is considered to be more proficient and powerful than other designs such as the three-level full factorial design, central composite design (CCD), and Doehlert design [19,20]. Moreover, degradation of membrane structure was also investigated by evaluation of membrane flux recovery rate (FRR), contact angle, pore size, and porosity as responses in the BBD.

## 2. Materials and methods

### 2.1. Chemical agents

Hydrogen peroxide (~30% wt.) was supplied from Merck (Darmstadt, Germany). The experiments were performed in a homemade stainless-steel reactor schematized in our previous study [16]. Nitrogen obtained from Linde gas (Turkey) was used to keep in-reactor pressure at a required level. A dead-end filtration system was used to obtain membrane experiments [21].

### 2.2. Membrane and cleaning procedure

A commercially available flat-sheet PES membrane (UP 150,000 Da MWCO, Microdyn-Nadir, Germany) was used in this study. UP150 is a hydrophilic and high-chemical-resistance ultrafiltration membrane. The pH range is from 0 to 14 with a maximum temperature of 95 °C. The new membrane coupons were soaked in ultrapure water overnight to ensure the removal of preservatives before use.

### 2.3. Hot peroxide oxidation method

The fouled membranes by MB dye were subjected to hot peroxide oxidation (HPO) using the experimental setup system given in the previous work [16]. The experiments were carried out in a homemade stainless-steel reactor, the reactor was heated by an external heater and temperature control was provided with a digital thermometer. Firstly, 150 mL of deionised water was put into the reactor and one fouled membrane coupon was placed in the water for each experiment. Then, depending on the experimental matrix applied, either a certain amount of H_2_O_2_ or no H_2_O_2_ was added, and the reactor was screwed off. Next, the reactor was pressurized to 30 bar using N_2_ gas. After that, the reactor was heated to a specific temperature and kept constant during a specified time (treatment time). The above-mentioned specific values of the experimental variables were performed according to the BBD schedule given in the following sections. After the treatment time was completed, the reactor was cooled and depressurized. Finally, the treated membrane was kept in deionised water for further analyses.

### 2.4. The BBD modelling

RSM allows observing changes in specific responses at specific levels of interest and quantitatively evaluating the behaviour of the tested area, using the correct model and different combinations of factors. The membrane cleaning process was further optimised using BBD using the Design-Expert program (version 9.0.6.2). The independent experimental variables, namely the concentration of H_2_O_2_ (*x*_1_), treatment time (*x*_2_), and temperature (*x*_3_) were explored as their effect on the process is significant. The process parameters and their respective ranges were determined based on preliminary experiments and relevant literature [15,19]. Besides, the temperature range that the membranes can withstand without deterioration has been taken into account. The three-level rotatable design matrix consisting of 17 runs was constructed by using the ranges of the independent variables (−1, 0, and +1) given in Table 1. The values of independent variables were chosen to test of UP150 membrane performance in the harsh environment conditions [20, 21]. In the BBD method, the number of experiments (*N*) is calculated using the following equation:


(1) $$N=K^{2}+K+C_{p}$$

where K and *C_p_* indicate the number of the independent variables and central points, respectively [22]. Twelve runs and 5 runs of the above-mentioned 17 runs indicate midpoints of the edge and the centre of the experimental design cube, respectively. The centre points enable the prediction of pure error as well as the calculation of the response at intermediate levels of a design and allow prediction of the system performance at any experimental point in the operating range [23]. The responses of the process, normalized permeability (J_w_/J_wo_), contact angle (°), porosity (%), and pore size (nm) followed in the BBD were represented by *Y*_1_, *Y*_2_, *Y*_3_, and *Y*_4_ respectively. Each response was calculated by taking the average of triplicated experiments.

## 3. Results and Discussion

### 3.1. Evaluation of the BBD models

The experimental and predicted results of 17 runs of the BBD of normalized permeability, contact angle, porosity, and pore size models are given in Table 2 along with their residual and leverage results. Leverages below 1 and lower residuals indicate the compatibility of the experimental and predicted values of a model. Thus, high agreements of the experimental and predicted values were obtained in all models according to the obtained residual and leverage results of all BBD models. In the normalized permeability model, the highest experimental and predicted permeability values were obtained in run 14 (2.34 J_w_/J_wo_ and 2.35 J_w_/J_wo_) and run 4 (1.34 J_w_/J_wo_ and 1.37 J_w_/J_wo_), respectively. Besides, the highest experimental and predicted contact angle values were obtained in run 4 as 67.19° and 67.08°, respectively, where the lowest experimental and predicted contact angle values were obtained in run 16 as 54.76° and 55.11°, respectively.

The highest experimental and predicted porosity values were obtained in run 14 as 86.06% and 84.99%, respectively, where the lowest experimental and predicted porosity values were obtained in run 8 as 61.06% and 60.78%, respectively. The highest experimental and predicted pore size values were obtained in run 16 as 160.73 nm and 161.52 nm, respectively, and the lowest experimental and predicted pore size values obtained in run 11 as 109.79 nm and 110.98 nm, respectively. The hypothetical equations provide the prediction of the response of the model in the working range and evaluation of the experimental variables on the response [15–17]. The second-order polynomial equations of normalized permeability, contact angle, porosity, and pore size models are given in Eq. 1, Eq. 2, Eq. 3, and Eq. 4, respectively. *Y*_1_, *Y*_2_, *Y*_3_, and *Y*_4_ demonstrate the obtained normalized permeability (J_w_/J_wo_), contact angle (°), porosity (%), and pore size (nm) in the related model, respectively. In Eq. 1,*x*_1_, *x*_3_ and *x*_1_*x*_2_, in Eq. 2,*x*_2_*x*_3_, *x*_1_^2^ and *x*_1_, in Eq. 3,*x*_1_*x*_3_, *x*_2_^2^ and *x*_1_^2^ and in Eq. 4,*x*_3_, *x*_2_^2^ and *x*_3_^2^ are the most effective model terms on the responses.


(2) $$Y_{1}=0.21x_{1}+0.067x_{2}+0.18x_{3}+0.18x_{1}x_{2}+0.15x_{1}x_{3}-0.028x_{2}x_{3}-0.15x_{1}^{2}-0.0076x_{2}^{2}+0.11x_{3}^{2}+1.85$$

(3) $$Y_{2}=-2.81x_{1}+1.59x_{2}+0.30x_{3}+0.34x_{1}x_{2}-0.31x_{1}x_{3}+3.54x_{2}x_{3}+3.07x_{1}^{2}-2.55x_{2}^{2}-0.0058x_{3}^{2}+57.39$$

(4) $$Y_{3}=-0.55x_{1}+2.11x_{2}+2.80x_{3}+2.02x_{1}x_{2}+8.61x_{1}x_{3}+1.84x_{2}x_{3}+3.25x_{1}^{2}-7.56x_{2}^{2}-1.86x_{3}^{2}+72.73$$

(5) $$Y_{4}=7.05x_{1}+3.86x_{2}+15.04x_{3}+1.70x_{1}x_{2}+3.67x_{1}x_{3}-2.73x_{2}x_{3}-6.63x_{1}^{2}+11.58x_{2}^{2}+9.44x_{3}^{2}+126.59$$

The results depicted that permeability, porosity, pore size, and contact angle were seriously affected by independent variables. H_2_O_2_ and temperature degraded the PES polymer depending on the treatment time and it caused to be obtained bigger pore sizes.

The suitability and adequacy of all applied BBD models can be evaluated using ANOVA results demonstrated in Table 3. High *F* value and low *p*-value (<0.05) indicate the significance of the model or the term. *p*-values were below 0.0001 in all models and F values were 41.19, 45.03, 82.29, and 85.90 in normalized permeability, contact angle, porosity, and pore size models, respectively. Therefore, it is seen that all models are significant in determining the effects of experimental variables on the responses where the pore size model is statistically more favourable. Besides, all terms of the normalized permeability model except *x*_2_*x*_3_ and *x*_2_^2^, *x*_1_, *x*_2_, *x*_3_, *x*_2_*x*_3_, *x*_1_^2^ and *x*_2_^2^ in the contact angle model, all terms of the porosity (except *x*_1_) and pore size (except *x*_1_*x*_2_) models are significant.

**Figure 1:**
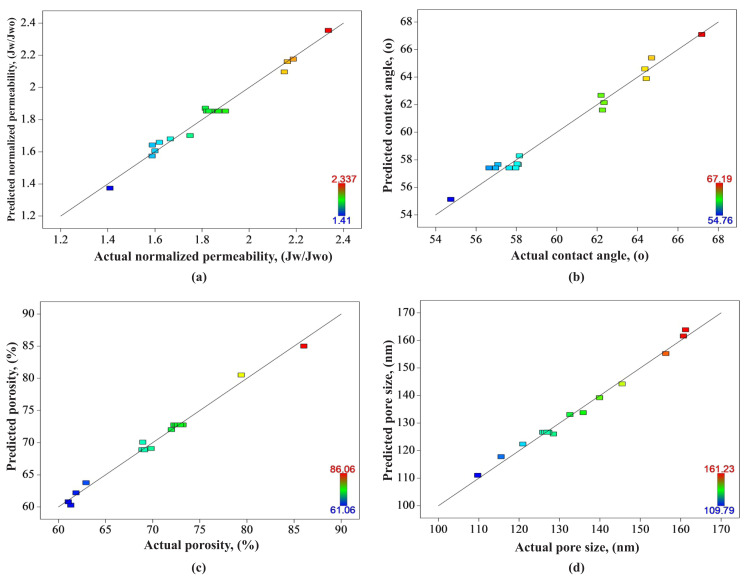
Correlation between the actual and predicted values of (a) normalized permeability, (b) contact angle, (c) porosity, and (d) pore size models.

Regression and correlation coefficients of all BBD models are displayed in Table 4. The coefficient of determination (R^2^) values were determined as 0.9815, 0.9830, 0.9906, 0.9910 in the normalized permeability, contact angle, porosity, and pore size models, respectively. Considering that the R^2^ value close to 1 indicates the relationship between variables and represents the percentage of variance explained by the model, the pore size model is the most favourable one [24]. Besides, the R^2^_adj_ value, which is a more useful good-fit parameter than R^2^ and used to compare different regression equations, was obtained as 0.9576, 0.9612, 0.9786, 0.9795 in the normalized permeability, contact angle, porosity, and pore size models, respectively. The correlation between experimental and predicted values can be measured by the closeness of R^2^_adj_ and R^2^_pre_ values [23]. Usually, the difference of less than 0.2 indicates a reasonable fit between them. In this case, considering the low difference (less than 0.2) between R^2^_adj_ and R^2^_pre_ values in all models indicates that all BBD models can be used safely with precision in obtaining predictive values [19]. This accordance was depicted in Figure 1.

In Figures 1a–1d, it can be seen that the points are well aligned along the 45-degree linear line. In accordance with the results in the regression coefficients given above, the fact that the points in Figure 1d are closer to the line shows that the actual and predicted values are so in agreement in the pore size model [19]. Also, a situation quite similar to the pore size model can be seen in the porosity model (Figure 1c). C.V. (%) value is the other indicator indicating the certainty of a model [23]. Herein, C.V. (%) values were quite close to each other in contact angle (1.19), porosity (1.41), and pore size (1.60) models, thus more favourable than the normalized permeability model (2.85).

**Figure 2:**
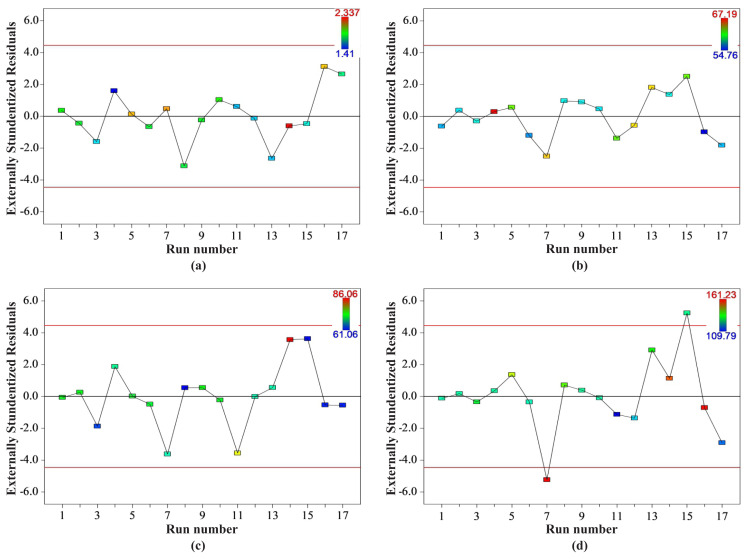
The plots of residuals vs experimental runs in (a) normalized permeability, (b) contact angle, (c) porosity, and (d) pore size models.

Figures 2a–2d demonstrate residuals versus experimental runs in normalized permeability, contact angle, porosity, and pore size models, respectively. Using this graphical evaluation, latent variables that could potentially affect the response in experiments can be controlled. Random scattering was observed in all graphs, and no run exceeding upper and lower control limits was observed in Figures 2a–2c. However, in Figure 2d, it was seen that run 7 and run 15 exceed the lower and upper control limits, respectively, and the other runs are generally in full compliance with the abovementioned limits. Also, the majority of the runs generally showed a distribution close to the 0.0 line in Figure 2b.

### 3.2. Combined effects of the experimental variables on the normalized permeability

**Figure 3:**
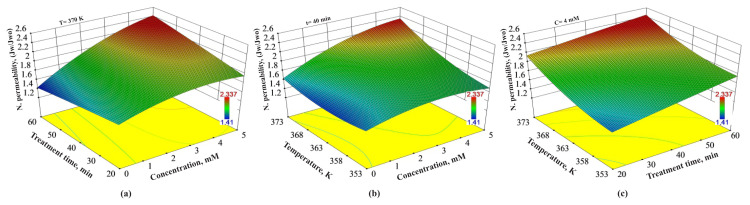
Combined effects of (a) treatment time and concentration of H_2_O_2_, (b) temperature and concentration of H_2_O_2_ and (c) temperature and treatment time on normalized permeability values.

The combined effects of treatment time, the concentration of H_2_O_2_ and temperature on the normalized permeability were demonstrated in Figures 3a–3c. Considering the red area demonstrating the high normalized permeability values, the higher values are seen to be squeezed into a very limited area in Figures 3a–3C, especially 3b. According to Figure 3a, high normalized permeability values were obtained in the moderate-high treatment time and a relatively high concentration of H_2_O_2_ values at 370 K constant temperature. However, at 40 min of treatment time, high normalized permeability values were obtained only at temperatures over 368 K and 2 mM of concentration of H_2_O_2_ (Figure 3b). Besides, reasonably high normalized permeability values were obtained at high-temperature values in almost all treatment times at a constant 4 mM of H_2_O_2_. For instance, increasing the treatment time from 20 min to 40 min and 60 min, respectively, increase the normalized permeability values from 2.0 J_w_/J_wo_ to 2.17 J_w_/J_wo_ and 2.32 J_w_/J_wo_, respectively, at a constant of 4 mM of H_2_O_2_ and 370 K of temperature.

### 3.3. Combined effects of the experimental variables on the contact angle

**Figure 4:**
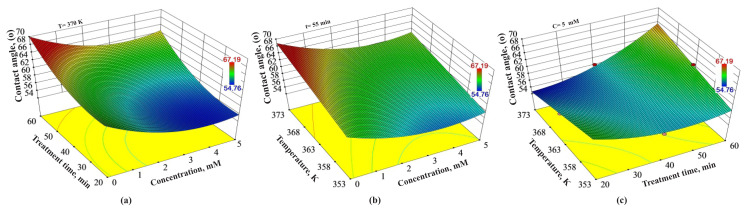
Combined effects of (a) treatment time and concentration of H_2_O_2_, (b) temperature and concentration of H_2_O_2_, and (c) temperature and treatment time on contact angle values.

Figure 4 demonstrates the combined effects of treatment time, the concentration of H_2_O_2_ and temperature on the contact angle. In Figure 4, compared to Figure 3, there is an excess of blue areas showing lower values than red areas showing higher values. According to Figure 4a, high contact angle values were obtained at low concentrations of H_2_O_2_ but high treatment time values at a constant 370 K of temperature. Besides, Figure 4b indicates that temperature must be set to the values higher than 363 K at 0–1 mM of concentration of H_2_O_2_ at 55 min of treatment time to obtain high contact angle values. For instance, at the constant concentration of H_2_O_2_ of 1 mM and treatment time of 55 min, altering the temperature from 353 K to 363 K and 373 K, respectively, provide the contact angles of 59.51°, 62.66°, and 65.80°. Figure 4c demonstrates that it is not possible to obtain the desired-height level of contact angle at any temperature and treatment time level when fixing the concentration of H_2_O_2_ to 5 mM.

### 3.4. Combined effects of the experimental variables on the porosity

**Figure 5:**
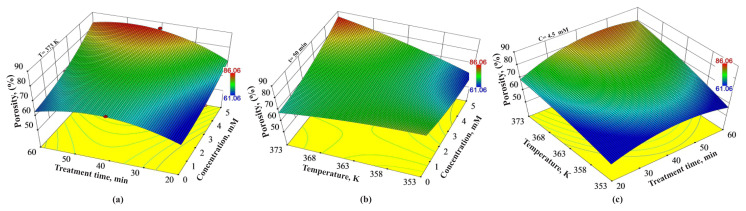
Combined effects of (a) treatment time and concentration of H_2_O_2_, (b) temperature and concentration of H_2_O_2_, and (c) temperature and treatment time on porosity values.

3D evaluation of the combined effects of experimental variables on the porosity is shown in Figure 5. According to Figure 5a, the porosity value of 61.06% could be increased to the levels of 86.06% at 373 K for medium-high treatment time and at high concentration levels. Also, Figure 5b reveals that both temperature and concentration have significant effects on the porosity. Thus, high porosity values could be obtained in the synergistic effect of the mentioned variables. Similarly, Figure 5c indicates that at a constant concentration of H_2_O_2_ of 4.5 mM, the porosity values increases at high temperature and medium-high treatment time. For instance, at a constant concentration of H_2_O_2_ of 4.5 mM and a temperature of 373 K, 69.08% of porosity value can be obtained. At the same conditions, only increasing the treatment time from 20 min to 40 min and 50 min, respectively, provide an increase in the porosity value to 82.20% and 83.09%, respectively. However, further increasing the treatment time to 60 min causes a decrease of porosity to 80.20%.

### 3.5. Combined effects of the experimental variables on the pore size

**Figure 6:**
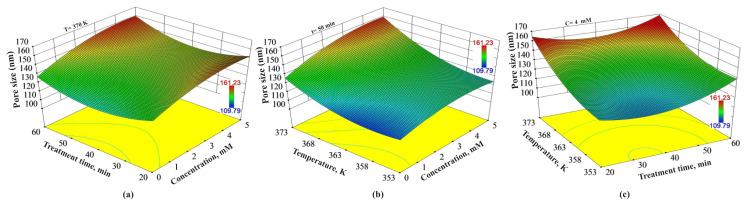
Combined effects of (a) treatment time and concentration of H_2_O_2_, (b) temperature and concentration of H_2_O_2_, and (c) temperature and treatment time on pore size values.

Figure 6 demonstrates the combined effects of treatment time, the concentration of H_2_O_2_, and temperature on pore size values. According to Figure 6a, the longer treatment time and the higher concentration of H_2_O_2_ increase the pore size at a constant temperature of 370 K. Also, Figure 6b reveals that at constant 50 min of treatment time low pore size values can be obtained at low-moderate temperature and low concentration values. Figure 6c demonstrates that lower pore size can be obtained from below of the border of the red area obtained at a constant concentration of H_2_O_2_ of 4 mM, high temperature and all treatment time values. At constant 4 mM of concentration of H_2_O_2_ and 373 K, the pore sizes increase from 155.12 nm to 159.12 nm by increasing the treatment time from 40 min to 50 min. Decreasing the temperature to 363 K at the same concentration of H_2_O_2_ provides 128.43 nm and 133.77 nm of pore sizes by increasing the treatment time from 40 min to 50 min. Moreover, further decreasing temperature to 353 K at a concentration of H_2_O_2_ 4 mM, provides 120.63 nm and 127.33 nm of pore size at the end of 40 min and 50 min of treatment time, respectively.

### 3.6. Optimum conditions of normalized permeability, contact angle, porosity and pore size models

**Figure 7:**
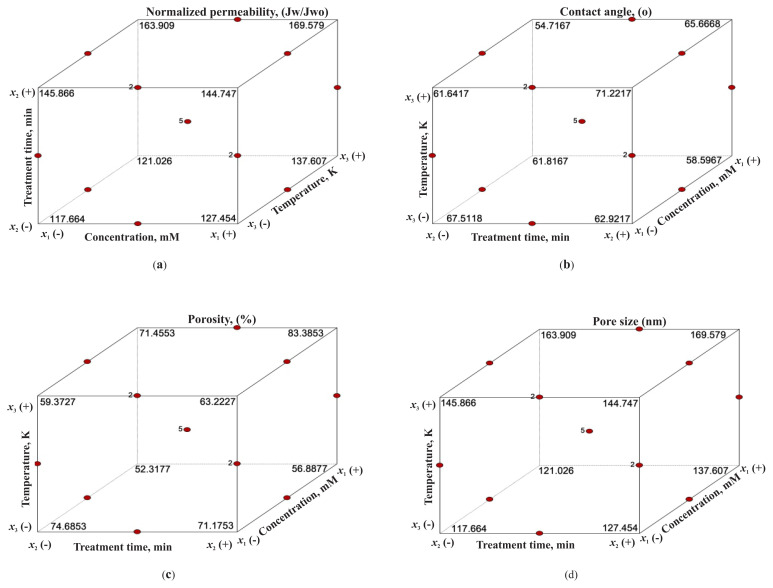
Cube plots of (a) normalized permeability, (b) contact angle, (c) porosity, and (d) pore size models.

Cube plots are very useful visuals that can be used to optimize the response obtained by the applied BBD model [25]. Figure 7 shows the predicted responses from the coded model for combinations of the lowest (−1) and highest (+1) levels of the three studied factors. Moreover, in a multiresponse system, these cube plots are effective both in determining the effect of variables on the responses and in determining the change in other responses for a particular level of a selected response. Figure 7a demonstrates that 117.67 J_w_/J_wo_ of normalized permeability value which can be obtained in the lowest level of all experimental factors can be increased to 145.87 J_w_/J_wo_ by only increasing the treatment time from the lowest level to the highest level. Also, at this condition, increasing the temperature to the highest level provides 163.91 J_w_/J_wo_ of normalized permeability. According to Figure 7b, 62.92° contact angle can be obtained at the lowest levels of temperature and concentration of H_2_O_2_ but the highest level of treatment time. At this conditions decreasing the treatment time to its lowest level, increasing the temperature to its highest level and increasing concentration of H_2_O_2_ to its highest level provides 67.51°, 71.22°, and 58.60° contact angle values in each case. Figure 7c demonstrates that 52.32% of porosity value can be increased to 71.46% by increasing temperature and further increased to 83.39% by further increasing the treatment time to its highest level. However, Figure 7c indicates that the pore size value is 169.58 nm at the conditions in which the porosity value is the highest. Thus, to decrease the pore size value from 169.58 nm to 137.61 nm, only decreasing temperature to the lowest value is sufficient. Furthermore, 117.66 nm of pore size can be obtained at the lowest value of all experimental variables. The previous study reported that H_2_O_2_ attack was believed to result in loss of the S=O links, i.e. a conversation of the -SO_2_ groups to charged – SO_3_ groups. Significant cracks on the membrane’s surface were observed for the pristine membranes exposed to a 5 wt.% of H_2_O_2_ solution due to the H_2_O_2_ and/or related radicals attacking the PES [26, 27].

## 4. Conclusion

In this study, the effects of H_2_O_2_ concentration, temperature, and treatment time were comprehensively investigated on filtration performance and physicochemical properties of the PES UP150 membrane. The degradation of the PES membrane was systematically investigated using response surface methodology (RSM). The effects of aging conditions were tested on membrane flux, contact angle, pore size, and porosity for decomposed membrane. H_2_O_2_ concentration with high temperature resulted in an obvious change in membrane permeability and pore size. The permeability results depicted that H_2_O_2_ aging caused chain scission of PES, leading to a significant increase in membrane pore size. Moreover, membrane hydrophilicity was decreased after the chain scission of PES. In general, the degree of membrane degradation caused by H_2_O_2_ exposure can be minimized by reducing the concentration of H_2_O_2_ and temperature.

## Figures and Tables

**Table 1:** The BBD design of the experimental variables of the membrane cleaning process.

Coded factors	Independent variables	Coded levels		
		−1	0	1
*x*_1_	Concentration of H_2_O_2_ (mM)	0	2.5	5
*x*_2_	Treatment time (min)	20	40	60
*x*_3_	Temperature (K)	353	363	373

**Table 2:** Experimental, predicted, residual, and leverage values of the BBD of normalized permeability, contact angle, porosity, and pore size models.

Run	Experimental variables			Normalized permeability (J_w_/J_wo_)				Contact angle (°)				Porosity (%)				Pore size (nm)			
	*x*_1_	*x*_2_	*x*_3_	Exp.	Pre.	Res.	L.	Exp.	Pre.	Res.	L.	Exp.	Pre.	Res.	L.	Exp.	Pre.	Res.	L.
1	2.5	40	363	1.87	1.85	0.018	0.20	56.98	57.39	−0.414	0.20	72.67	72.73	−0.062	0.20	126.35	126.59	−0.242	0.20
2	2.5	40	363	1.83	1.85	−0.022	0.20	57.65	57.39	0.256	0.20	72.97	72.73	0.238	0.20	126.95	126.59	0.358	0.20
3	5	20	363	1.62	1.66	−0.038	0.75	58.16	58.27	−0.112	0.75	62.95	63.74	−0.794	0.75	132.63	133.03	−0.399	0.75
4	0	60	363	1.41	1.37	0.038	0.75	67.19	67.08	0.113	0.75	69.85	69.06	0.794	0.75	127.06	126.66	0.399	0.75
5	5	60	363	2.16	2.16	0.004	0.75	62.35	62.14	0.213	0.75	72.00	71.99	0.006	0.75	145.53	144.15	1.376	0.75
6	2.5	40	363	1.82	1.85	−0.032	0.20	56.65	57.39	−0.744	0.20	72.27	72.73	−0.462	0.20	125.87	126.59	−0.722	0.20
7	2.5	60	373	2.19	2.18	0.013	0.75	64.70	65.38	−0.675	0.75	68.97	70.05	−1.081	0.75	161.23	163.79	−2.564	0.75
8	2.5	60	353	1.82	1.87	−0.054	0.75	58.04	57.69	0.350	0.75	61.06	60.78	0.281	0.75	139.95	139.16	0.789	0.75
9	2.5	40	363	1.84	1.85	−0.012	0.20	57.98	57.39	0.586	0.20	73.24	72.73	0.508	0.20	127.38	126.59	0.788	0.20
10	2.5	40	363	1.90	1.85	0.048	0.20	57.71	57.39	0.316	0.20	72.51	72.73	−0.222	0.20	126.41	126.59	−0.182	0.20
11	0	40	353	1.59	1.57	0.017	0.75	62.20	62.66	−0.463	0.75	79.42	80.50	−1.075	0.75	109.79	110.98	−1.188	0.75
12	0	20	363	1.60	1.60	−0.004	0.75	64.37	64.58	−0.212	0.75	68.88	68.89	−0.006	0.75	120.95	122.33	−1.376	0.75
13	0	40	373	1.59	1.64	−0.051	0.75	64.44	63.88	0.563	0.75	69.15	68.86	0.288	0.75	135.89	133.73	2.165	0.75
14	5	40	373	2.34	2.35	−0.017	0.75	58.10	57.64	0.463	0.75	86.06	84.99	1.075	0.75	156.35	155.16	1.188	0.75
15	2.5	20	353	1.67	1.68	−0.013	0.75	62.27	61.60	0.675	0.75	61.33	60.25	1.081	0.75	128.54	125.98	2.564	0.75
16	2.5	20	373	2.15	2.10	0.054	0.75	54.76	55.11	−0.350	0.75	61.88	62.16	−0.281	0.75	160.73	161.52	−0.789	0.75
17	5	40	353	1.75	1.70	0.051	0.75	57.09	57.65	−0.562	0.75	61.88	62.17	−0.287	0.75	115.57	117.74	−2.165	0.75

*x*_1_: Concentration of H_2_O_2_, (mM); *x*_2_: Treatment time, (min), *x*_3_:Temperature, (K); Exp.: Experimental, Pre. Predicted, Res.: Residual, L.: Leverage

**Table 3:** ANOVA results of the BBD of normalized permeability, contact angle, porosity, and pore size models.

Models →	Normalized permeability				Contact angle				Porosity				Pore size			
Source	Sum of squares	Mean Square	*F* value	*p*-value prob > *F*	Sum of squares	Mean Square	*F* value	*p*-value prob > *F*	Sum of squares	Mean Square	*F* value	*p*-value prob > *F*	Sum of squares	Mean Square	*F* value	*p*-value prob > *F*
Model	1.008	0.112	41.19	<0.0001	206.474	22.942	45.03	<0.0001	720.97	80.108	82.29	<0.0001	3540.16	393.35	85.90	<0.0001
x_1_	0.353	0.353	129.70	<0.0001	63.281	63.281	124.21	<0.0001	2.43	2.431	2.50	0.1581	397.48	397.48	86.81	<0.0001
x_2_	0.036	0.036	13.35	0.00814	20.225	20.225	39.70	0.0004	35.45	35.448	36.41	0.0005	119.51	119.51	26.10	0.0014
x_3_	0.261	0.261	95.96	<0.0001	0.720	0.720	1.41	0.2733	62.55	62.552	64.26	<0.0001	1810.52	1810.52	395.40	<0.0001
x_1_x_2_	0.135	0.135	49.52	0.0002	0.469	0.469	0.92	0.3692	16.32	16.322	16.77	0.0046	11.53	11.53	2.52	0.1566
x_1_x_3_	0.086	0.086	31.56	0.0008	0.378	0.378	0.74	0.4174	296.70	296.701	304.78	<0.0001	53.88	53.88	11.77	0.0110
x_2_x_3_	0.003	0.003	1.13	0.3226	50.197	50.197	98.53	<0.0001	13.542	13.542	13.91	0.0074	29.76	29.76	6.50	0.0381
x_1_^2^	0.090	0.090	32.94	0.0007	39.664	39.664	77.86	<0.0001	44.55	44.549	45.76	0.0003	185.14	185.14	40.43	0.0004
x_2_^2^	0.000	0.000	0.09	0.7729	27.470	27.470	53.92	0.0002	240.95	240.949	247.51	<0.0001	564.76	564.76	123.34	<0.0001
x_3_^2^	0.051	0.051	18.86	0.0034	0.000	0.000	0.00	0.9873	14.52	14.524	14.92	0.0062	375.14	375.14	81.93	<0.0001
Residual	0.019	0.003	-	-	3.566	0.509	-	-	6.81	0.973	-	-	32.05	4.58	-	-
Lack of fit	0.015	0.005	4.60	0.0873	2.333	0.778	2.52	0.1966	6.23	2.078	14.30	0.0132	30.69	10.23	30.04	0.0033
Pure error	0.004	0.001	-	-	1.234	0.308	-	-	0.58	0.145	-	-	1.36	0.34	-	-

**Table 4:** Regression and correlation coefficients of the BBD of normalized permeability, contact angle, porosity, and pore size models.

Regression coefficients	Normalized permeability	Contact angle	Porosity	Pore size
Standart deviation	0.052	0.714	0.987	2.140
Mean	1.83	60.04	69.83	133.36
C.V. %	2.85	1.19	1.41	1.60
PRESS	0.24	39.25	100.64	493.18
R^2^	0.9815	0.9830	0.9906	0.9910
Adjusted R^2^	0.9576	0.9612	0.9786	0.9795
Predicted R^2^	0.7636	0.8131	0.8617	0.8619
Adequate precision	24.53	21.86	32.69	32.18
